# RARE‐LacZ Mice as a Model to Study of Retinoic Acid Signaling in Alzheimer’s Disease

**DOI:** 10.1002/alz.092980

**Published:** 2025-01-03

**Authors:** Ashly Hindle, Shane Smith, Matthew Hernandez, Adam Baker, Jake Strickland, Jocelyn Medina, J. Josh Lawrence

**Affiliations:** ^1^ Texas Tech University Health Sciences Center, Lubbock, TX USA; ^2^ Texas Tech University, Lubbock, TX USA

## Abstract

**Background:**

Disrupted balance between amyloidogenic and non‐amyloidogenic pathways leads to cognitive decline in Alzheimer’s disease (AD). Evidence suggests vitamin A (VA) supplementation favors the non‐amyloidogenic pathway through upregulation of α‐secretase. Originally used to map embryonic retinoic acid (RA) signaling, RARE‐LacZ mice possess multiple LacZ genes controlled by retinoic acid response elements (RAREs). We crossed RARE‐LacZ mice with AD mouse models to determine their suitability for quantitative studies into the effects of VA on dentate gyrus (DG) RA signaling and learning in AD.

**Methods:**

Relative LacZ gene copy ratio was determined by qPCR of LacZ in gDNA, normalized to ultra‐conserved region 329. Dietary intervention compared VA supplemented (20 IU/g) AIN‐93M to standard AIN‐93M (4 IU/g VA). Mice were tested at postnatal day (P)125 via water T‐maze (WTM, 9 simple discrimination, 9 reversal trials). PFA‐fixed sections (40µm) were immunostained for LacZ, doublecortin, and/or calbindin, confocally imaged, and analyzed using ImageJ.

**Results:**

RARE‐lacZ mice were first crossed with C57BL/6J, ‐NJ, and CD1 mice (wildtype strains of J20, hAβ‐KI, and RARE‐LacZ mice, respectively). The relative LacZ gene copy ratio was ∼2.6:1 between RARE‐LacZ mice (N = 12) and crosses (N = 34). Values for 32/34 offspring fell within ±50% of the mean. Strain affected latency to platform on WTM during simple discrimination and reversal (N = 11‐12, p<0.05, Friedman), however total distance traveled was unaffected suggesting intact learning in all backgrounds. In WT strains, hippocampal LacZ immunoreactivity was localized to a subset of mature doublecortin‐negative, calbindin‐positive DG granule cells, appearing higher on C57BL/6J and ‐NJ than CD1 backgrounds. Offspring from J20^±^ AD and RARE‐LacZ mice exhibited impaired learning (N = 16, p<0.05, Kolmogorov‐Smirnov). No significant difference in behavior between supplemented (20 IU/g) and standard (4 IU/g) VA was observed in AD mice. However, DG LacZ signal was completely silenced in 7/32 mice evaluated by immunostaining.

**Conclusions:**

RARE‐LacZ mice appear to have behavioral and genetic characteristics appropriate for testing VA‐mediated interventions in AD models. RA signaling is prominent in mature DG cells associated with successful reversal learning. Although reversal of AD‐related learning deficits by VA supplementation was not observed, testing AD mice on a VA deficient (0.4 IU/g) diet is planned.

